# Infectiousness to sand flies of a cat naturally infected with *Leishmania infantum* at the moment of diagnosis and after three different courses of treatment

**DOI:** 10.1590/S1984-29612025006

**Published:** 2025-02-03

**Authors:** Mariana Dantas da Silva, Andrea Cristina Higa Nakaghi, Fredy Galvis-Ovallos, João Augusto Franco Leonel, Geovanna Vioti, Eunice Aparecida Bianchi Galati, Nayara Cristina de Oliveira Fazolato, Julia Pinho Martins, Trícia Maria Ferreira de Sousa Oliveira

**Affiliations:** 1 Programa de Pós-graduação em Saúde Pública, Faculdade de Saúde Pública, Universidade de São Paulo – USP, São Paulo, SP, Brasil; 2 Universidade de Sorocaba – UNISO, Sorocaba, SP, Brasil; 3 Departamento de Epidemiologia, Faculdade de Saúde Pública, Universidade de São Paulo – USP, São Paulo, SP, Brasil; 4 Programa de Pós-graduação em Epidemiologia e Saúde Única, Faculdade de Medicina Veterinária e Zootecnia, Universidade de São Paulo – USP, São Paulo, SP, Brasil; 5 Departamento de Medicina Veterinária, Faculdade de Zootecnia e Engenharia de Alimentos, Universidade de São Paulo – USP, Pirassununga, SP, Brasil

**Keywords:** Feline leishmaniosis, visceral leishmaniosis, marbofloxacin, miltefosine, allopurinol, Leishmaniose felina, leishmaniose visceral, marbofloxacina, miltefosina, alopurinol

## Abstract

In this study, an evaluation was made of three treatments against feline leishmaniosis (FeL) and their impacts on the transmission of *Leishmania infantum* to its vector, *Lutzomyia longipalpis*. A cat with clinical signs of FeL was examined and *L*. *infantum* diagnosed. Subsequently, the cat was subjected to xenodiagnosis and *L. infantum* detected in the vectors. The cat was then treated with three different drugs and the clinical improvement and parasite transmissibility to the vector were evaluated. Promastigotes were observed in 21/52 female sandflies (40.38%) in a xenodiagnosis prior to the treatments. Clinical signs persisted after the first treatment with marbofloxacin, and the cat remained positive in serological, molecular, and parasitological tests. Therefore, the cat was treated with miltefosine but remained sick and tested positive. A second xenodiagnosis was performed a month after treatment with miltefosine, and promastigotes were observed in 5/9 females (55.55%). Lastly, the cat was treated with allopurinol, which led to good clinical improvement, but it remained positive, and a final xenodiagnosis revealed *Leishmania* in 2/29 (6.89%) females. The results showed that only treatment with allopurinol produced a good clinical response, but none of the treatments succeeded in eliminating *L. infantum* infection or preventing transmission to the vector.

## Resumo

Neste estudo, três tratamentos contra a leishmaniose felina (FeL) e seus impactos na transmissão de *Leishmania infantum* ao vetor, *Lutzomyia longipalpis,* foram avaliados. Um gato com sinais clínicos de FeL foi examinado e *L. infantum* detectada. Submetido ao xenodiagnóstico, verificou-se a transmissão de *L. infantum* aos vetores. O gato foi então tratado com três medicamentos diferentes e a melhora clínica e transmissibilidade do parasito ao vetor foram avaliadas. Foram observadas leishmanias em 21/52 fêmeas de flebotomíneos (40,38%) no xenodiagnóstico antes dos tratamentos. Os sinais clínicos persistiram após o primeiro tratamento com marbofloxacina, e o gato permaneceu positivo em testes sorológicos, moleculares e parasitológicos. Na sequência, o gato foi tratado com miltefosina, mas continuou doente e com testes positivos. Um segundo xenodiagnóstico foi realizado um mês após o tratamento com miltefosina. Observaram-se promastigotas em 5/9 fêmeas (55,55%). Por fim, o gato foi tratado com alopurinol, o que o levou a uma boa melhora clínica, mas sem cura parasitológica, e um xenodiagnóstico final detectou promastigotas em 2/29 (6,89%) fêmeas. Os resultados mostraram que somente o tratamento com alopurinol produziu uma boa resposta clínica, mas nenhum dos tratamentos conseguiu eliminar a infecção por *L. infantum* ou impedir sua transmissão ao vetor.

## Introduction

In Brazil, *Leishmania infantum* infection in cats was first described in 2004 (Savani et al., 2004) and since then, *Leishmania* infection has been described in cats throughout the country, with *L. infantum* being the most common species (Nascimento et al., 2022).

The most frequent clinical signs of feline leishmaniosis (FeL) are skin lesions, enlarged lymph nodes and ocular lesions, regardless of the species of *Leishmania*. (Silveira et al., 2015; Pennisi et al., 2015). The diagnostic tests currently employed to detect *Leishmania* infection in cats are the same as those available for dogs. A combination of serological and molecular techniques is the most recommended approach, including sequencing of samples in areas with more than one endemic *Leishmania* species (Silva et al., 2023). With regard to the treatment of infected cats, the scientific literature offers case reports and treatment protocols based on clinical veterinary experience, using drugs prescribed for dogs, some of them off-label, and the best results in clinical resolution are provided by allopurinol (Fernandez-Gallego et al., 2020; Garcia-Torres et al., 2022).

Xenodiagnosis is the ideal technique to determine whether an infected host can naturally transfer the pathogen to a potential vector (Maia & Campino, 2011; Quinnell & Courtenay, 2009). To date, reports and descriptions of the treatment of cats with FeL are scanty, and in none of the reported cases was a treated cat’s ability to infect the vector evaluated. The aim of this study was to evaluate the infectivity of colonized females of *L. longipalpis* fed on a cat naturally infected with *L. infantum* after three different treatment protocols. Unfortunately, the study was limited to just one cat, as 184 cats were tested in a previous active search and only one was positive.

## Material and Methods

### Patient history

A male mixed breed cat from the municipality of Votorantim (23° 32' 49” S 47° 26' 16” O), São Paulo state, Brazil was treated at the Veterinary Hospital of the University of Sorocaba. The cat’s owner brought the animal to the hospital due to dermatological lesions, which had persisted for several months. The cat shared the environment with other cats, but none of them showed these or any other clinical signs.

### Feline leishmaniosis diagnosis

#### Samples

Samples of blood, serum, popliteal lymph node and bone marrow aspirates were taken for diagnostic tests.

#### Serological diagnosis

An indirect immunofluorescence antibody test (IFAT) was carried out as described by Vides et al. (2011), to detect anti-*Leishmania* spp. antibodies. Feline retroviruses were tested using a Antibody Test kit (IDEXX Laboratories).

#### Parasitological diagnosis

Popliteal lymph node and bone marrow aspirates were placed on glass slides and stained with Diff-Quick. The smears were analyzed under an optical microscope (100X) to detect amastigote forms of *Leishmania*.

#### Molecular diagnosis

DNA was extracted from blood, popliteal lymph node and bone marrow samples using a DNeasy^®^ Blood & Tissue kit (QUIAGEN, USA) and amplified by PCR according to Rodgers et al. (1990). A second ITS1 PCR was carried out (El Tai et al., 2000) and products were subjected to Sanger sequencing. The electropherograms of the sequences were checked using Chromas software. Then, the consensus was generated using BioEdit and compared using the Basic Local Alignment Search Tool (BLAST).

### Treatment protocols

Following the diagnosis of FeL, the cat was treated monotherapically with a daily oral dose of 5 mg/kg marbofloxacin (Marbopet^®^, CEVA), for 30 days. After almost a month without any medication, miltefosine (Milteforan™, Virbac) at 2 mg/kg, once a day, was administered orally for 28 days. One month after the miltefosine monotherapy, due to the persistence of clinical signs, the cat was treated with allopurinol at 20 mg/kg orally once a day for 120 days.

### Clinical follow-up

Before each treatment and at 10-day intervals until the end (day 0, day 10, day 20, day 30), the clinical and laboratory profiles were assessed. Body score and temperature, mucous membrane, color and dermatological signs, hydration, splenomegaly, and lymphadenopathy were observed through clinical examination. Red blood cells, hemoglobin, hematocrit, white blood cells and platelets were assessed using an automated hematology analyzer. The serum biochemical profile, including serum proteins (albumin and globulin), creatinine, urea, ALT and alkaline phosphatase, were assessed using an automated UV spectrophotometer. Urinalysis and the urine protein/creatinine ratio were examined. In addition, on day 30, blood, serum, lymph node and bone marrow samples were collected and subjected to the above described FeL diagnostic test.

### Xenodiagnosis

Xenodiagnosis was carried out before the cat started treatment (xenodiagnosis 1), then one month after the treatments with marbofloxacin (xenodiagnosis 2) and miltefosine (xenodiagnosis 3), and on the last day of treatment with allopurinol (xenodiagnosis 4).

#### Phlebotomine sandfly specimens

*Lutzomyia longipalpis* females, 3 to 5 days old, reared in a closed colony were used. The sandflies were kept as described by Killick-Kendrick & Killick-Kendrick (1991) and modified as described by Galvis-Ovallos et al. (2017), at the Entomology Laboratory, University of São Paulo School of Public Health (USP). Males phlebotomine were added to stimulate blood feeding (Vioti et al., 2022).

#### Exposure to sandflies

The cat was anesthetized with zolazepam hydrochloride, tiletamine hydrochloride (11 mg/kg) and tramadol (2 mg/kg), and then placed in an individual 50 x 30 x 30 cm nylon cage. Males and females *L. longipalpis* were released into the nylon cage and the cat exposed to bites for 60 minutes.

#### Dissection of female sandflies

Dissection was carried out under a stereomicroscope at 60X magnification, immediately after the death of the females, as described by Diniz et al. (2014). Females surviving up to 120 hours after the blood meal were euthanized by freezing at 4°C for 6 minutes. The intestine was analyzed under an optical microscope (400X) to investigate the presence of promastigotes. The intensity of promastigote infection was expressed using the qualitative approach described by Travi et al. (2001), where (-) means no promastigotes; (+) weak infection with 1-50 present, (++) moderate infection with 51-100, (+++) strong infection with 101-200, and (++++) intense infection with >201 promastigotes. After observation under the microscope, the coverslip was lifted with tweezers and washed with absolute alcohol in a sterile 1.5 ml microtube. The same procedure was carried out on the slide containing the female intestine, with all the material transferred to the same microtube. Samples from the intestines of female sandflies were stored at -20°C until DNA extraction.

#### DNA extraction and molecular assay of female sandfly samples

DNA was extracted individually from the intestines of *L. longipalpis* females, as described by Bruford et al. (1998) and modified by Galvis-Ovallos et al. (2017). The in-house protocol consisted of macerating dissected sandflies in a cell lysis solution (20mM EDTA 0.5M, 50mM Tris-HCL, 117mM NaCl and 1% SDS), followed by the addition of proteinase K (10 mg/mL). The proteins were denatured by adding 4M ammonium acetate, and the DNA was precipitated by adding absolute alcohol followed by 70% alcohol. The DNA was resuspended in 50 μl of ultrapure water.

The quality of the extraction was checked according to Lins et al. (2008) and PCR carried out as described by Pita-Pereira et al. (2005). To identify *Leishmania* spp. on the sandflies gut, the same aforementioned PCR was carried out.

## Results

### Clinical presentation, confirmation of *L. infantum* infection, and first xenodiagnosis

**Figure 1:**
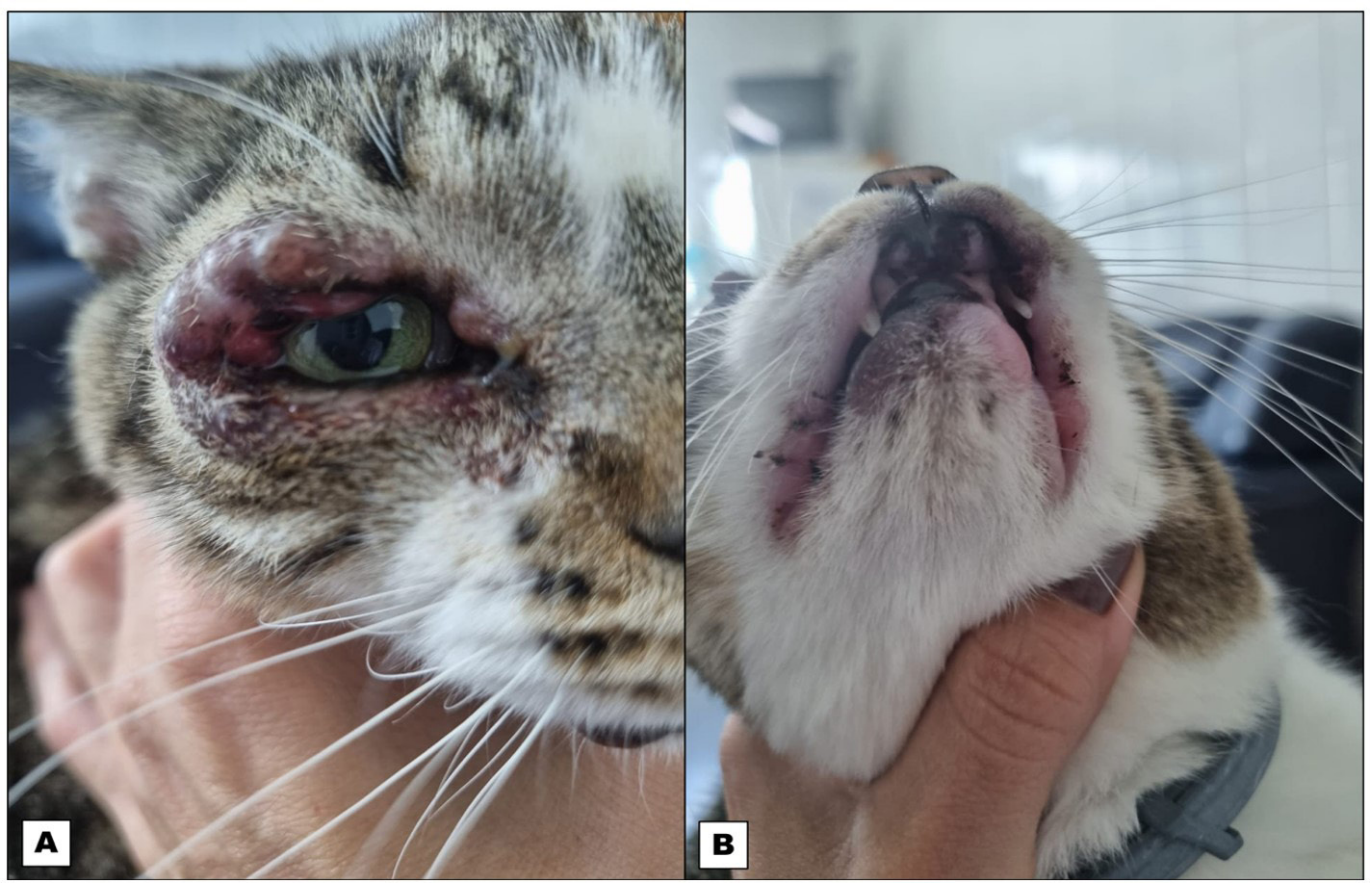
Granulomatous lesions on the eyelids (A) and papule granulomatous lesions on the lower lip (B) associated with *Leishmania infantum* in an infected cat before treatment.

The cat had nodules on the eyelids, oral nodules on the upper and lower lips, and abdominal distension (Figure 1). Palpation revealed splenomegaly and lymphadenopathy. No other abnormalities (body score, temperature, mucous membranes, and hydration) were observed in a clinical examination. The hematological parameters were all normal for the species and the serum biochemical tests showed normal concentrations of urea, creatinine, alkaline phosphatase, ALT, cholesterol, and triglycerides. However, the globulin concentration was high (5.3 g/dL).

The cat was negative for feline retroviruses and positive for *Leishmania* spp. PCR in blood, lymph node and bone marrow samples. ITS1 sanger sequencing confirmed *L. infantum* infection. The IFAT test was positive with a titer of 1:10,240. In addition, amastigote forms of *Leishmania* sp*.* were found in lymph nodes and bone marrow by cytology.

After FeL diagnosis, the cat was subjected to the first xenodiagnosis (xenodiagnosis 1), prior to any treatment. To this end, 72 female sandflies were released to feed on the cat, 52 of which became engorged and were dissected. *L*. *infantum* promastigotes were detected in 40.38% (21/52) of *L. longipalpis* females, which showed 13 weak (+), 2 moderate (++) and 6 intense (++++) infection and *Leishmania* spp. kDNA was detected in 71.42% (35/49) of tested female sandflies.

### Treatment 1: clinical and laboratory profiles, and second xenodiagnosis after therapeutic protocol with marbofloxacin

Clinical signs showed a slight improvement on days 10, 20 and 30 of marbofloxacin treatment. Although they did not disappear, a reduction was observed in the granulomatous lesions on the eyelids and lips. Splenomegaly and lymphadenopathy were still detected during clinical examinations on all the follow-up days. Normal mucous membranes, body score, temperature and hydration status were observed during all the days of treatment.

Serum biochemistry tests showed normal concentrations of urea, creatinine, alkaline phosphatase, ALT, cholesterol, and triglycerides. However, the globulin concentration was high at the end of treatment (6.0 g/dL).

No abnormalities were found in the urinalysis, and the urinary protein/creatinine ratio showed normal parameters before, during and after marbofloxacin treatment.

The cat’s blood, lymph node and bone marrow samples remained positive by PCR, and its lymph nodes and bone marrow by cytology. The IFAT titer was the same as before treatment, i.e., 1:10,240.

After 30 days of treatment with marbofloxacin, the cat underwent a xenodiagnosis (xenodiagnosis 2). However, due to the repellent effect of a commercial imidacloprin/flumethrin collar (removed 10 days before the xenodiagnosis), the female *L. longipalpis* sandflies did not feed on the cat’s blood. Moreover, all the sandfly individuals died within 24 hours (Table 1).

**Table 1:** Parasitological examination of *Lutzomyia longipalpis* females engorged on blood from a *Leishmania infantum* infected cat during xenodiagnosis.

**TIME**	**INFECTION INTENSITY**			
	**(+)**	**(++)**	**(+++)**	**(++++)**
	**(presence of 1-50 promastigotes)**	**(presence of 51-100 promastigotes)**	**(presence of 101-200 promastigotes)**	**(presence of > 201 promastigotes)**
Xenodiagnosis 1	13/21 (61.9%)	2/21 (9.5%)	0	6/21 (28.5%)
Xenodiagnosis 2	0	0	0	0
Xenodiagnosis 3	2/5 (40%)	2/5 (40%)	1/5 (20%)	0
Xenodiagnosis 4	2/2 (100%)	0	0	0

### Treatment 2: clinical and laboratory profiles, and third xenodiagnosis after therapeutic protocol with miltefosine

After ten days of miltefosine treatment, the lymph nodes, spleen and liver were slightly less swollen than in the first examination. At the end of treatment, the size of the lymph nodes continued to decrease, but no difference was detected in the skin lesions on the lips and eyelids. Again, all the clinical parameters were normal during physical examinations before, during and at the end of treatment. As before, no changes were observed in hematological tests, serum biochemistry or urine parameters, except for serum globulin concentration (6.2 d/dL) and IFAT titer (1:5,120), which remained high even after miltefosine treatment

The cat remained positive in all the PCR, lymph node and bone marrow cytology samples. However, the parasite load showed a decrease during miltefosine medication, especially in bone marrow cytology, compared to the period before treatment.

One month after concluding the treatment with miltefosine and 15 days without wearing a commercial imidacloprin/flumethrin collar, another xenodiagnosis was carried out (xenodiagnosis 3). Of the 49 *L. longipalpis* females exposed to the treated cat, 100% (49/49) of them fed on the cat’s blood, but their mortality rate in the first 24 hours was high, leaving only 9 female phlebotomines for dissection. Five *L. longipalpis* females 55.55% (5/9) had flagellated forms of *L. infantum* in the midgut, with 2 showing a weak intensity of infection (+), 2 with moderate infection (++) and 1 with strong infection (+++) (Table 1). *Leishmania* spp. kDNA was detected in one female (11.1%; 1/9).

### Treatment 3: clinical and laboratory profiles, and fourth xenodiagnosis after therapeutic protocol with alopurinol

**Figure 2:**
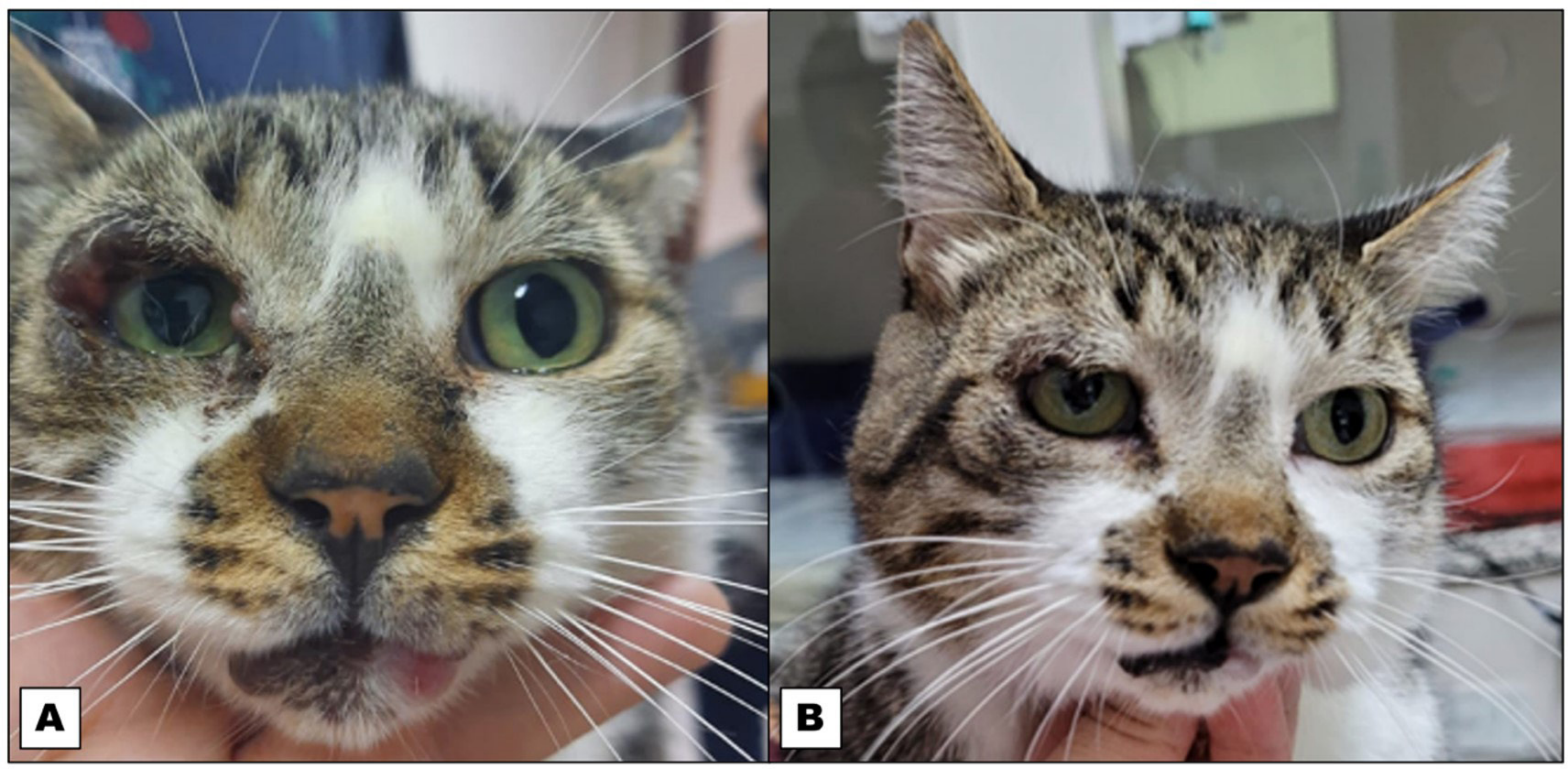
Granulomatous lesions on the eyelids and papule granulomatous lesions on the lower lip associated with *Leishmania infantum* infection before treatment (A), and after treatment with alopurinol (B).

After 30 days without medication, the cat’s clinical signs persisted, so it was treated with allopurinol. Allopurinol treatment was effective in reducing the eye and lip lesions within a few days of treatment and after 120 days. The granulomatous lesion on the lip disappeared, and the size of the lymph nodes was normal upon palpation at the end of the treatment protocol (Figure 2). A physical examination revealed no abnormalities.

No abnormalities other than hyperglobulinemia (6.9 g/dL) were found in the hematological and biochemical tests. Although allopurinol is an inhibitor of the xanthine oxidase enzyme, no xanthine stone sediment was detected in the urine, and the urine test and urinary protein/creatinine ratio were normal. All the samples taken from the cat remained positive by PCR and parasitology, with *L. infantum* amastigotes detected in the lymph nodes and bone marrow. The animal also remained positive by IFAT, with a high titer of 1: 10,240.

Prior to a final xenodiagnosis (xenodiagnosis 4), the cat had spent 32 days without wearing a commercial imidacloprin/flumethrin collar. Sixty-five female *L. longipalpis* were used to feed on the cat. Forty-seven or 70.3% (47/65) became engorged with blood. Twenty-nine or 61.7% female sandflies were dissected (29/47), and 6.89% (2/29) were weakly infected with *L. infantum* promastigotes (Table 1). *Leishmania* spp. kDNA was detected in the midgut of a parasitologically positive female 50% (1/2) and in a pool of 18 female sandflies that had fed on the cat’s blood but could not be dissected.

## Discussion

In this study, a cat with FeL and all diagnostic tests confirming *L. infantum* infection was treated with three drugs commonly used to treat canine leishmaniosis (CanL) in Brazil: marbofloxacin, miltefosine and allopurinol. A slight clinical improvement was observed after treatment with marbofloxacin and miltefosine, although no parasitological cure was achieved. Miltefosine is a drug approved to treat CanL now in Brazil (Santos Nogueira et al., 2019) and although the effects of quinolones on *Leishmania* are still unknown, there is some evidence that marbofloxacin can be used to treat kidney patients (Pineda et al., 2017).

Treatment with allopurinol resulted in a significant clinical improvement, but again, the cat remained positive for all the FeL diagnostic tests. In a review on the subject, Garcia-Torres et al. (2022) reported that the mean survival time of treated cats was statistically superior to that of untreated cats, and monotherapy with oral allopurinol for at least 6 months showed a good clinical response and increased survival in most cats. It is important to note that allopurinol is a known inhibitor of the xanthine oxidase enzyme, and prolonged treatment can cause urinary xanthine stones, which was not observed during this cat’s five months of treatment.

Since the first reports of the transmission of *L. infantum* to vectors through naturally infected cats, these feline species have been in the spotlights of the VL cycle (Maroli et al., 2007; Silva et al., 2010; Vioti et al., 2022).

In this study, a xenodiagnosis was carried out before starting the treatment protocols. The cat infected with *L. infantum* was able to transmit the parasite at a higher rate to the phlebotomine *L. longipalpis*. As with the treatment of FeL, there is a lack of studies evaluating the transmission of *L. infantum* from treated cats to vectors. In this research, we performed a xenodiagnosis after each therapeutic protocol, and although there was an apparent reduction in the transmission of the protozoan to the vectors after allopurinol treatment, the cat remained infectious.

The control of *Leishmania* spp. infection in cats is also still poorly understood and studied. No vaccines are available and currently only imidacloprin/flumethrin collars can be used, due to the sensitivity of cats to pyrethroids. According to Brianti et al. (2017), collars with this type of formulation proved effective in reducing cases of *L. infantum* infection in cats. Although an evaluation of the efficacy of imidacloprin/flumethrin collars was outside the scope of this study, our results suggest a residual effect of the product, since 100% mortality of *L. longipalpis* sandflies was observed when sandflies were exposed to the cat without a collar 10 days earlier (xenodiagnosis 2), and the sandfly mortality rate exceeded 80% when the collar was removed 15 days earlier (xenodiagnosis 3). Furthermore, no repellent and/or insecticidal effect was observed only more than 30 days after the collar was removed.

## Conclusions

Our results demonstrate that a cat with FeL treated with marbofloxacin, miltefosine and allopurinol showed clinical improvement only after five months of allopurinol monotherapy. However, even after all the treatments, the animal remained serologically, parasitologically and molecularly positive and infectious for the vector *L. longipalpis*. Veterinarians should be aware of *L. infantum* infection in the clinical routine of cats, and repellent collars appear to be a good alternative for controlling this infection in this species.
